# Stochastic vagus nerve stimulation affects acute heart rate dynamics in rats

**DOI:** 10.1371/journal.pone.0194910

**Published:** 2018-03-28

**Authors:** Steven W. Lee, Kanchan Kulkarni, Elizabeth M. Annoni, Imad Libbus, Bruce H. KenKnight, Elena G. Tolkacheva

**Affiliations:** 1 Department of Biomedical Engineering, University of Minnesota, Minneapolis, MN, United States of America; 2 LivaNova, PLC (Cyberonics, Inc.), Houston, TX, United States of America; Universita degli Studi di Bologna, ITALY

## Abstract

Vagus nerve stimulation (VNS) is an approved therapy for treatment of epilepsy and depression. While also shown to be promising in several preclinical and clinical studies to treat cardiovascular diseases, optimal therapeutic stimulation paradigms are still under investigation. Traditionally, parameters such as frequency, current, and duty cycle are used to adjust the efficacy of VNS therapy. This study explored the effect of novel stochastic VNS (S-VNS) on acute heart rate (HR) dynamics. The effect of S-VNS was evaluated in Sprague Dawley rats by comparing the acute HR and HR variability (HRV) responses to standard, periodic VNS (P-VNS) across different frequencies (FREQs, 10–30 Hz). Our results demonstrate that both S-VNS and P-VNS produced negative chronotropic effects in a FREQ-dependent manner with S-VNS inducing a significantly smaller drop in HR at 10 Hz and 20 Hz compared to P-VNS (p<0.05). S-VNS demonstrated a FREQ-dependent drop in the SD1/SD2 ratio, a measure of HRV, which was absent in P-VNS, suggesting that S-VNS may acutely modulate the nonlinear relationship between short- and long-term HRV. In conclusion, S-VNS is a novel stimulation procedure that may provide different physiological outcomes from standard P-VNS, as indicated by our analysis of HR dynamics. Our study provides a rationale for further detailed investigations into the therapeutic potential of S-VNS as a novel neuromodulation technique.

## Introduction

In recent years, there has been an emergence of interest in using neuromodulation techniques to enhance or suppress activity of the nervous system for the treatment of various pathological conditions, including but not limited to systemic inflammation, obesity, spinal cord injuries, and neurological disorders. One such therapy is vagus nerve stimulation (VNS), which was approved by the FDA as a clinical therapy for the treatment of refractory epilepsy in 1997 and for the treatment of medication-resistant depression in 2005 [1]. As a result, VNS has become one of several non-pharmacological treatment options to control epileptic seizures worldwide.

The benefits of using VNS to treat cardiovascular diseases, including cardiac arrhythmias, heart failure (HF), hypertension, and myocardial ischemia, have been extensively explored in past decades, with several studies suggesting the therapy to be both safe and potentially effective as a long-term cardiac therapy [2–5]. However, randomized clinical trials evaluating VNS in chronic HF patients have shown discrepant findings [6]. While the INOVATE-HF and NECTAR-HF trials failed to demonstrate significant improvements, both ANTHEM-HF and the subsequent extension study (ENCORE) yielded favorable and encouraging results including reduced HF symptoms and improved ventricular function [5,7–9]. In addition to different inclusion and exclusion criteria of HF patients, the different choice of stimulation parameters is another attributable factor for these mixed results.

Traditionally, the therapeutic intensity of VNS is selected to demonstrate engagement of the autonomic nervous system (typically via observed changes in heart rate (HR)) [10]. The stimulation parameters used to treat cardiac diseases, however, are highly variable depending on different models and studies [2,4,11,12]. Hence, there is an ongoing search for the optimal settings that will maximize the benefits of VNS. This can include the traditional approach of varying the stimulation frequency, current/voltage, and pulse width [12,13], as well as developing novel stimulation paradigms, such as the recently developed “microburst” VNS [10]. Typically, chronic periodic VNS (P-VNS) is applied, and its stimulation profile consists of administering periodic, repetitive electrical pulses with the level of stimulation (i.e. intensity) determined by a selected frequency, pulse duration, pulse amplitude, and duty cycle which all can be adjusted via radiofrequency communication link using a proprietary programming computer. In this study, we propose and evaluate an innovative stimulation technique: stochastic VNS (S-VNS). The version of S-VNS used in this study delivers electrical impulses to the nerve according to a Gaussian distribution of stimulation frequencies, instead of a constant frequency used in standard, P-VNS.

It is known that the cardiac beat-to-beat time intervals vary stochastically due to the inherent variability in HR (HRV). This variability can be attributed to many physiological factors including the influence of circadian rhythms, respiratory rhythms, temperature regulations, and others [14]. In fact, it has been extensively reported that both increased HR and loss of HRV are associated with increased mortality in HF patients and are candidate markers for patients at risk of sudden cardiac death [15,16]. An abundance of literature supports that acute P-VNS effectively decreases HR and improves HRV via the extensive innervation of the vagus nerve into the sinoatrial (SA) and atrioventricular (AV) nodes [17,18]. Even though the exact mechanisms responsible for the coupling between VNS and HR and HRV remains unclear, it is postulated that it is mediated through muscarinic receptor activation via the neurotransmitter acetylcholine (ACh) released at parasympathetic nerve terminals [19]. However, while P-VNS may effectively modulate HR dynamics, there may exist a ‘physiological compensation mechanism’ in which the vagus nerve and the central-peripheral nervous system can adapt to the periodicity of the stimulation, a phenomena which can be similar to pharmacological tolerance [20–22]. We propose that S-VNS might help to avoid this potential adaptation, and therefore, the goal of our study is to evaluate the influence of acute S-VNS therapy on instantaneous HR and HRV by using *in-vivo* rat hearts, and compare it to the standard P-VNS.

## Materials and methods

Sprague-Dawley rats (n = 8, 250–400 g, Charles River Laboratories, Wilmington, MA) were used in this study. All experiments were performed in accordance with the guidelines set forth by the National Institutes of Health Guide for the Care and Use of Laboratory Animals and were approved by the University of Minnesota Institutional Animal Care and Use Committee (IACUC).

### Vagus nerve bipolar cuff electrode implantation

Rats were anesthetized with isoflurane (5% for induction and 1.5% for maintenance). After hair shaving and skin cleaning, aseptic technique was used to make a ventral midline incision in the neck, and the skin and muscles were retracted. After identifying and isolating the right cervical vagus nerve and common carotid artery bundle, a custom 1.5 mm diameter helical lead bipolar cuff electrode (Cyberonics, USA) was implanted around the bundle. The right vagus nerve was chosen for this study because it primarily innervates the atria and the sinoatrial node, and thus, right-side stimulation may induce significant change in cardiac rhythm [23]. The electrode was then connected to a battery-driven, constant current stimulator (Stimulus Isolator, model A385, World Precision Instruments, USA). At the end of the study, the anesthetized rats were euthanized by thoracotomy followed by heart explantation.

### VNS protocols and study design

Both P-VNS and S-VNS were continuously delivered for 2 minutes at a pulse duration of 500 µsec and an output current of 1.0 mA [2–4]. The same number of stimuli was used for both P-VNS and S-VNS.

P-VNS was delivered at different frequencies (FREQ) of 10, 20, or 30 Hz that were kept constant throughout the 2 minutes of stimulation.

S-VNS was used with stochasticity (STOCH) of 10% or 20%. Three values of mean FREQ (i.e. 10, 20, and 30 Hz) were used similar to P-VNS, and STOCH (using a Gaussian distribution) was incorporated as:

$${FREQ}_{STOCH}=\frac{FREQ}{1\pm [FREQ*\delta (STOCH)]}$$

where δ(STOCH) is a random number with a mean of zero and a standard deviation of STOCH/FREQ. A custom LabView program was used to generate δ(STOCH) for each pulse.

**Fig 1:**
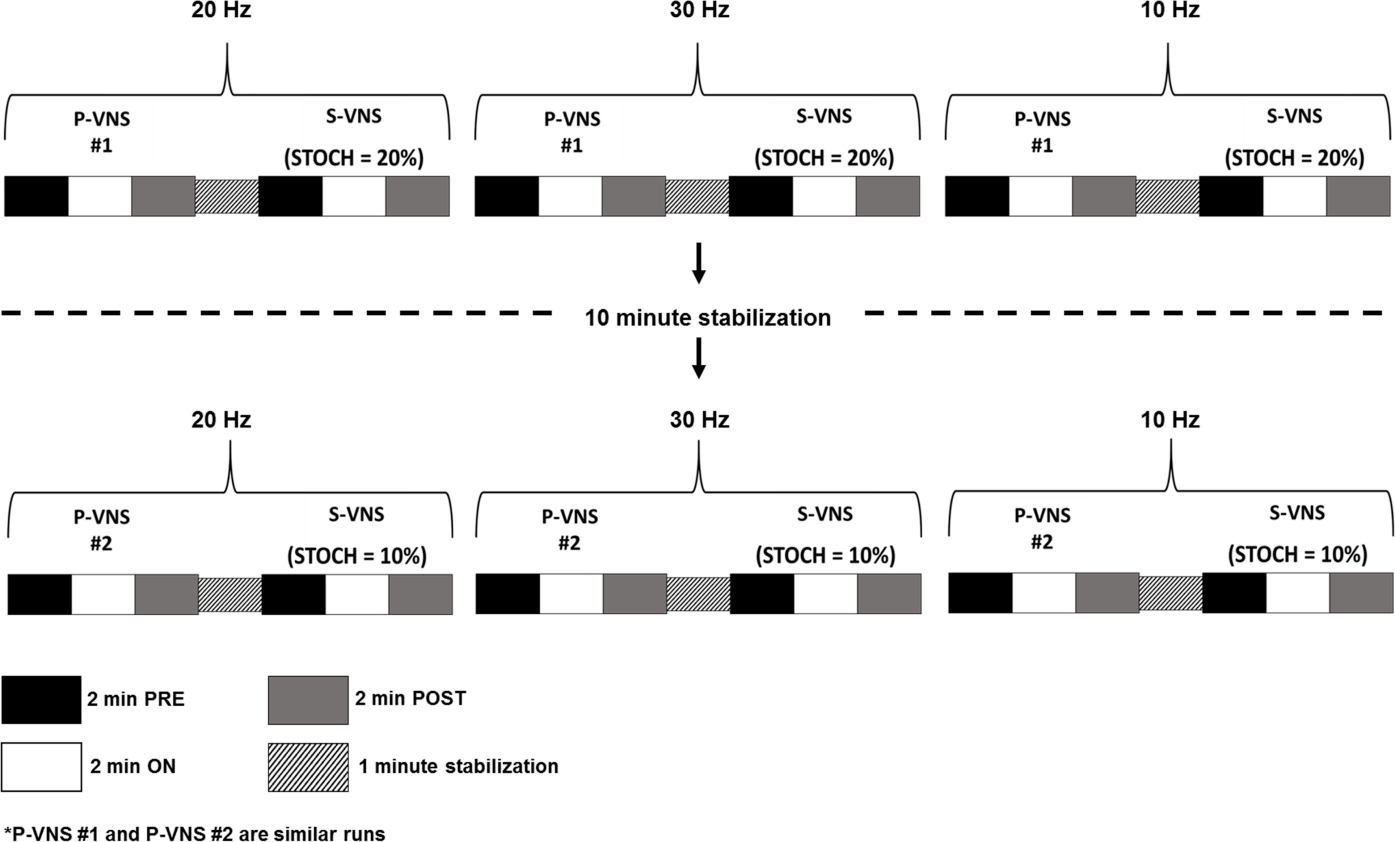
Detailed schematic of the experimental VNS protocol. Standard P-VNS or S-VNS with different degree of stochasticity (STOCH, 10% and 20%) was administered across different frequencies (FREQ, 20, 30, and 10 Hz) with stabilization times between protocols and conditions. P-VNS, periodic vagus nerve stimulation; S-VNS, stochastic vagus nerve stimulation; STOCH, stochasticity; **PRE**, baseline recording; **ON**, continuous VNS; **POST**, recovery.

The detailed study design is shown in Fig 1. Briefly, the vagus nerve was first stimulated at FREQ = 20 Hz, starting with P-VNS (P-VNS #1) followed by S-VNS with STOCH = 20% after a 1-minute stabilization interval. After completion, a similar run was conducted for a FREQ = 30 Hz which was followed by a run at FREQ = 10 Hz. After a 10-minute stabilization interval, both P-VNS (P-VNS #2) and S-VNS with STOCH = 10% runs were conducted in the FREQ order of 20, 30 and 10 Hz. Each protocol for a certain FREQ consisted of the following stimulation:

1. 2 minutes of baseline recording before VNS (**PRE**)
2. 2 minutes of continuous VNS (**ON**)
3. 2 minutes of recovery after VNS (**POST**)

### Data analysis for in-vivo ECG recordings

Electrocardiogram (ECG) was recorded from each anesthetized rat (IX-ECG-12, iWorx, USA). ECG electrodes were placed subcutaneously into the limbs. For both P-VNS and S-VNS, **PRE**, **ON**, and **POST** ECG data were recorded for 2 minutes. The *in-vivo* ECG recordings were used to quantify changes in the HR and HRV throughout the study using Kubios HRV 2.0 software [24]. Noisy data segments and ectopic beats were excluded and steady-state data after adjustment of the HR to acute VNS were used for the analysis. Hence, all runs of **PRE, ON,** and **POST** for all animals consisted of ~1-minute and 40 seconds interval for analysis.

To account for variations in baseline HR, the chronotropic effect of VNS was determined by calculating a relative change in HR, *ΔHR*_*ON*_, as the following:

$$\Delta {HR}_{ON}=\left(\frac{{HR}_{ON}-{HR}_{PRE}}{{HR}_{PRE}}\right)*100\%$$

where HR_ON_ and HR_PRE_ are the mean HR during the VNS **ON** and **PRE** periods, respectively.

After the termination of VNS, we assessed the recovery of HR to HR_PRE_ baseline value. Therefore, the HR post-VNS ratio (*HR*_*POST Ratio*_) was calculated as

$${HR}_{POST\;Ratio}=\left|\frac{\Delta {HR}_{POST}}{\Delta {HR}_{ON}}\right|$$

and

$$\Delta {HR}_{POST}=\left(\frac{{HR}_{POST}-{HR}_{ON}}{{HR}_{ON}}\right)*100\%$$

where HR_POST_ is the mean HR during the VNS **POST** period. A *HR*_*POST Ratio*_ = 1 means that the HR_POST_ returned to the baseline HR_PRE_ value. Whereas, if *HR*_*POST Ratio*_ < 1 (or > 1), then the HR_POST_ was less (or greater) than the baseline HR_PRE_ value.

To quantify HRV, Poincaré plots were used to provide a two-dimensional representation of the correlation between successive RR intervals, as described previously [24]. Briefly, the spatial distribution of all points was fitted to an ellipse with the center coinciding with the average RR interval. The two dispersions (perpendicular (SD1) and parallel (SD2) to the line of identity y = x) and their ratio, SD1/SD2, were then calculated. Previously, it was established that SD1 is a measure of the short-term HRV, while SD2 provides an assessment of both the short- and long-term HRV [24,25].

### Statistical analysis

All data are presented as means ± standard error. Statistical analyses were performed using Origin 9.1 software (Northampton, MA). Statistical comparisons were performed using a one-way ANOVA with repeated measures: 1) FREQ-dependent effect of P-VNS and S-VNS, 2) between S-VNS and P-VNS; 3) between the two P-VNS protocols, and 4) FREQ-dependent effect on SD1/SD2 for **PRE**, **ON**, and **POST**. A one sample *t-*test was performed to assess if the mean of the *HR*_*POST Ratio*_ is equal to one. Values of *P <* 0.05 were considered to be statistically significant. If appropriate, post hoc comparisons with Tukey’s test were performed, with *P*-values corrected for repeated comparisons.

## Results

**Fig 2:**
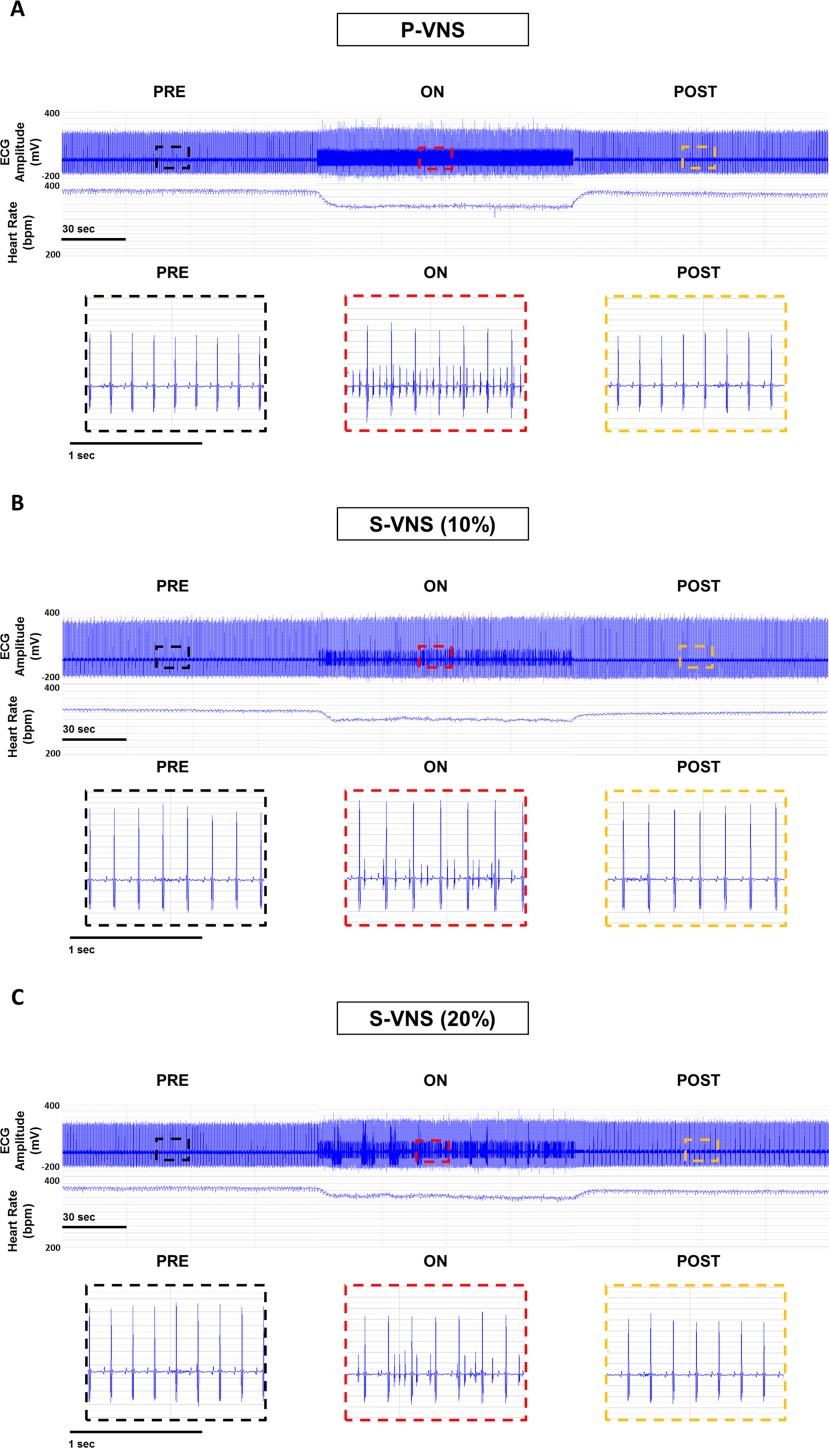
ECG recordings and corresponding HR responses. Representative segments of ECG recordings and corresponding heart rate (HR) response for an anesthetized rat for **PRE**, **ON**, and **POST** during **(A)** P-VNS, **(B)** S-VNS (10%), and **(C)** S-VNS (20%) of the right cervical vagus nerve. Zoomed-in snapshots of **PRE** (black), **ON** (red), and **POST** (yellow) highlights VNS artifacts during stimulation. Here, VNS was continuously delivered at 20 Hz, 500 µs pulse width, and 1.0 mA for 2 minutes.

Fig 2 shows typical examples of ECG traces during P-VNS (Panel A) and S-VNS for STOCH = 10% and 20% (Panels B and C, respectively). Note the presence of small spikes on the ECG traces during the VNS **ON** periods corresponding to VNS stimulation artifacts, and the VNS-evoked decrease in HR. Furthermore, note the periodic, repetitive pattern of the VNS artifacts during P-VNS, and the absence of periodicity of the artifacts during S-VNS.

### Frequency-dependent chronotropic effects of P-VNS and S-VNS

**Fig 3:**
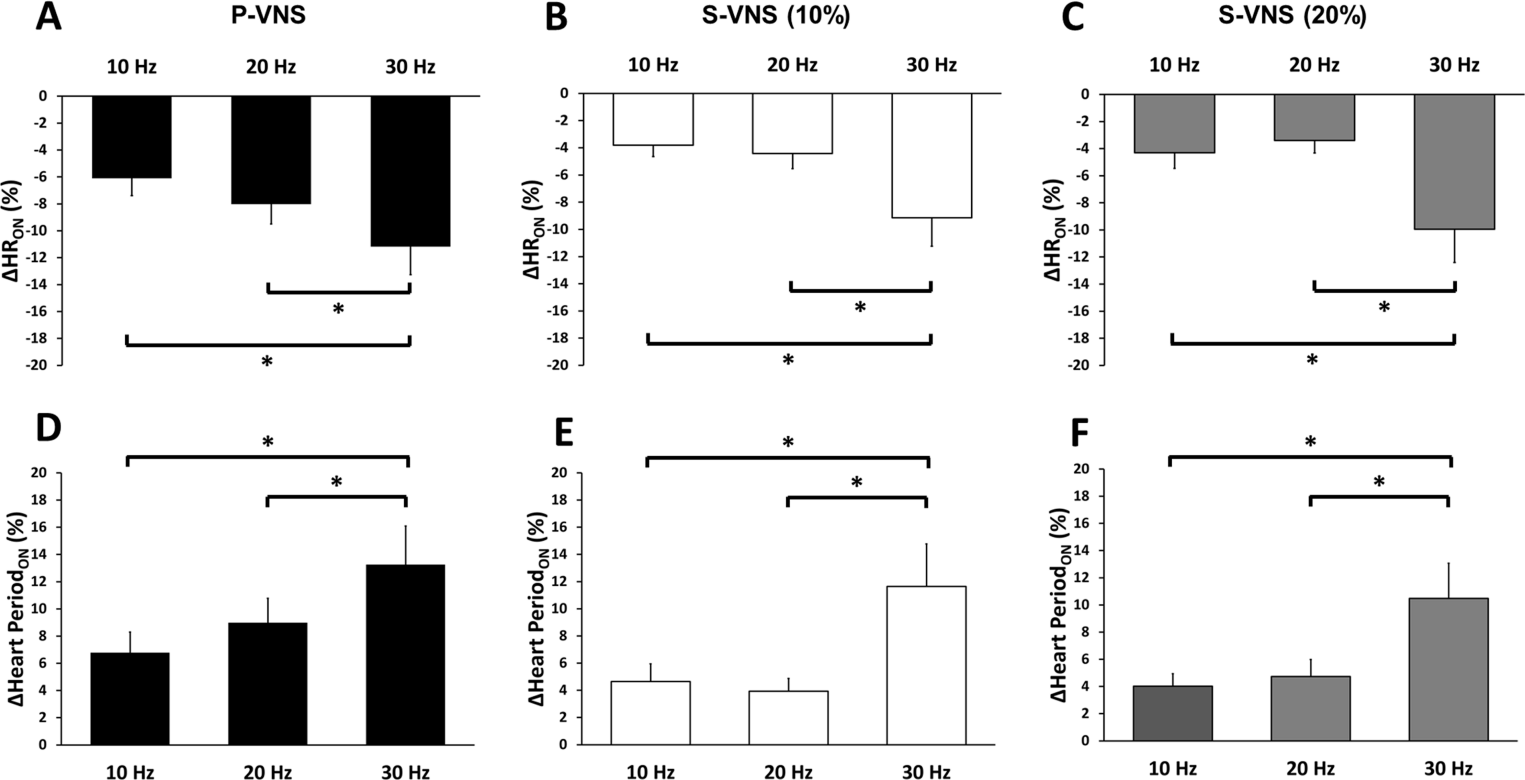
Effects of VNS FREQ on HR and heart period. Mean percent drop in HR for **(A)** P-VNS, **(B)** S-VNS (10%), and **(C)** S-VNS (20%) at different FREQ. Mean percent drop in Heart Period for **(D)** P-VNS, **(E)** S-VNS (10%), and **(F)** S-VNS (20%) at different FREQ. Note data reported here for P-VNS is the mean and SEM of the average of P-VNS #1 and P-VNS #2 protocols (n = 8). (*p < 0.05).

The FREQ-dependent chronotropic effects of P-VNS and S-VNS are shown in Fig 3. Note that the reported mean and SEM of the P-VNS protocols were averages of the P-VNS protocols (i.e., mean of the average of P-VNS #1 and P-VNS #2 protocols, n = 8). Relative changes in HR, *ΔHR*_*ON*_, in response to VNS across FREQ ranging from 10 to 30 Hz are shown for P-VNS (Panel A) and S-VNS (Panels B, C). For both P-VNS and S-VNS (both STOCH = 10% and 20%), no significant reduction in HR was observed as FREQ was increased from 10 Hz to 20 Hz. However, further increase of FREQ to 30 Hz produced a significant drop in HR.

It has been shown in several species such as dogs [26] and rats [27] that VNS FREQ is linearly related to heart period, while the relation to HR is nonlinear. To account for this possibility, we also calculated the relative changes in heart period for both VNS protocols. Fig 3 (Panels D-F) indicate that FREQ-dependent chronotropic effect for heart period is similar to the one for HR (compare to Fig 3, Panels A-C).

### Effects of STOCH on HR and HRV

**Fig 4:**
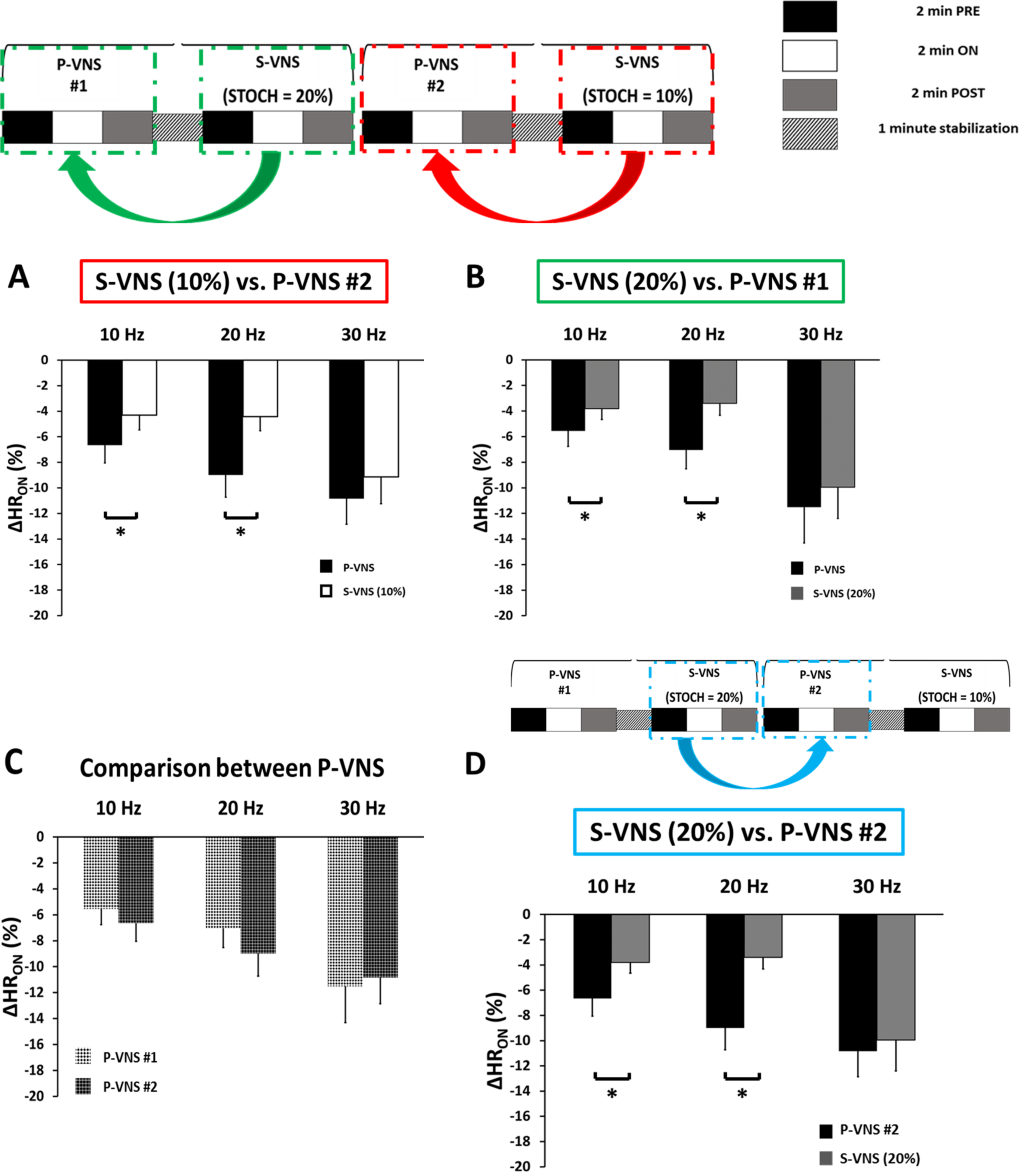
Effects of STOCH on the chronotropic effects of VNS. Mean percent drop in HR for **(A)** S-VNS (10%) and **(B)** S-VNS (20%) being compared to its immediate preceding P-VNS protocol at different FREQ. **(C)** Comparison of mean relative drop in HR between P-VNS protocols and **(D)** S-VNS (20%) and its subsequent P-VNS protocol at different FREQ. (*p < 0.05).

The relative changes in HR, *ΔHR*_*ON*_, in response to 10% (Panel A) and 20% S-VNS (Panel B), respectively, compared to its immediate preceding P-VNS at different FREQs are shown in Fig 4. Overall, S-VNS had significantly smaller negative chronotropic effect relative to P-VNS at FREQ = 10 and 20 Hz. At 30 Hz, however, this reduced effect was eliminated as all the protocols exhibited similar relative drop in HR. Moreover, note that both P-VNS runs, P-VNS #1 and P-VNS #2, exhibited similar drop in HR at different FREQs, as indicated in Panel C, suggesting the stability of the experimental conditions over time. To investigate whether the order of application of P-VNS and S-VNS protocols contribute to the effects shown in Panels A and B, 20% S-VNS was compared to its subsequent P-VNS #2 at different FREQs. Fig 4D demonstrates similar effect in the relative change in HR responses when compared to Panels A and B, indicating that the effect is attributed to the S-VNS protocol itself, but not the different permutation of the two stimulation protocols.

**Fig 5:**
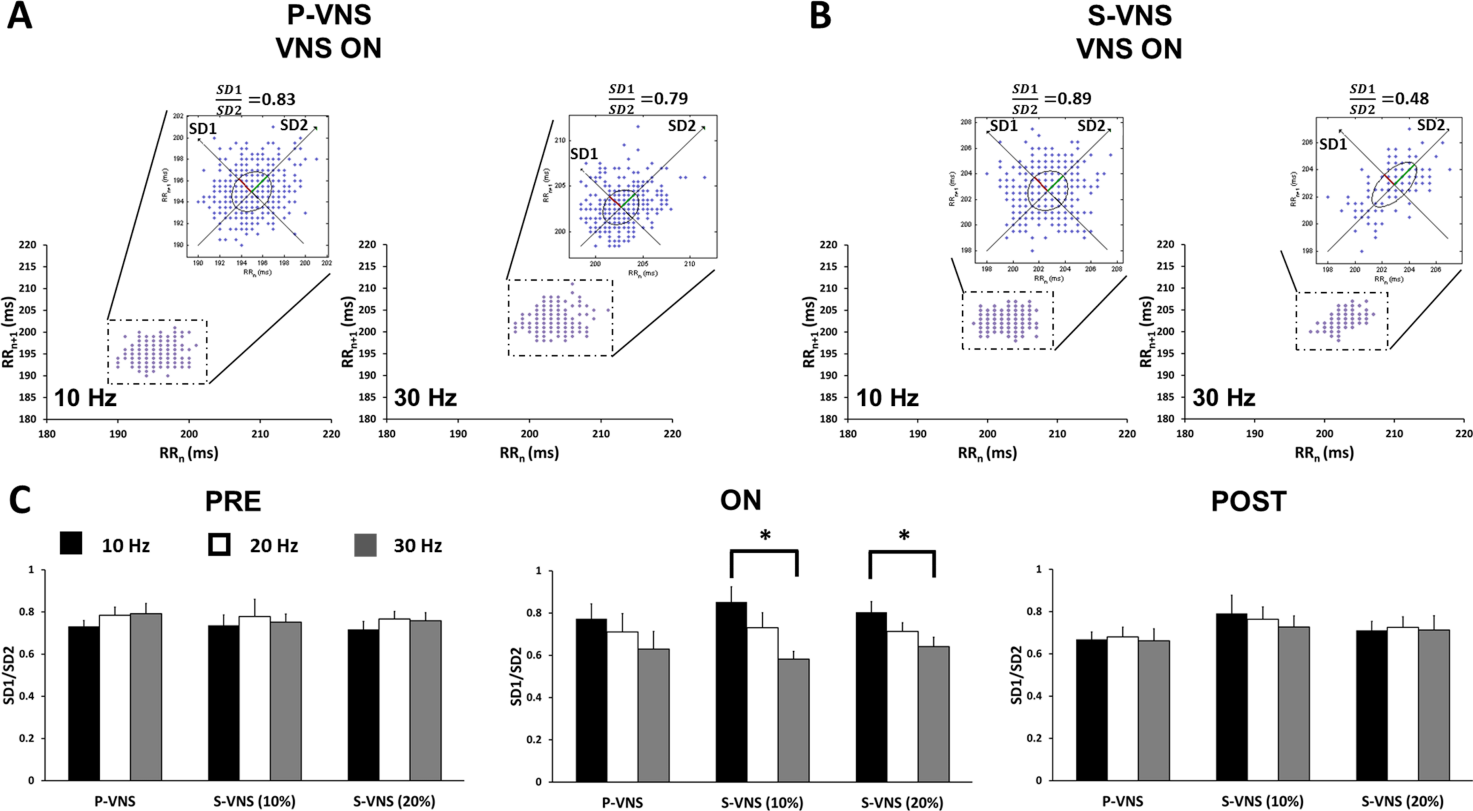
Effects of VNS on HRV using Poincaré analysis. Representative Poincaré plots of **(A)** P-VNS and **(B)** S-VNS (10%) during VNS stimulation (**ON**) at 10 Hz and 30 Hz demonstrating the elliptical fitting of the beat-distribution cloud and the standard deviation of short-term (SD1) and long-term (SD2) variability. **(C)** Mean SD1/SD2 ratio for **PRE**, **ON** and **POST** across different FREQ. (*p < 0.05).

Representative Poincaré plots of both P-VNS (Panel A) and S-VNS (Panel B) and their quantitative assessment, SD1/SD2 (Panel C) are shown in Fig 5. While our results suggest that S-VNS did not significantly affect SD1/SD2 when compared to P-VNS (as indicated in Fig 5C), we observed a FREQ-dependent effect. As shown in Fig 5C, for P-VNS protocol, the SD1/SD2 values were similar for all FREQs for **PRE**, **ON**, and **POST** periods. Similarly, no significant difference in SD1/SD2 values was observed for S-VNS for **PRE** and **POST** periods. However, for VNS **ON** period, S-VNS showed significant reduction in SD1/SD2 values between FREQ = 10 Hz and 30 Hz. In addition to Poincaré analysis, we also evaluated HRV as a ratio of the standard deviation of RR interval (SDRR) to mean RR interval (mean RR), as described previously [28]. From this approach, no significant difference was observed for all FREQs for **PRE**, **ON**, and **POST** periods for all VNS protocols (S1 Fig).

**Fig 6:**
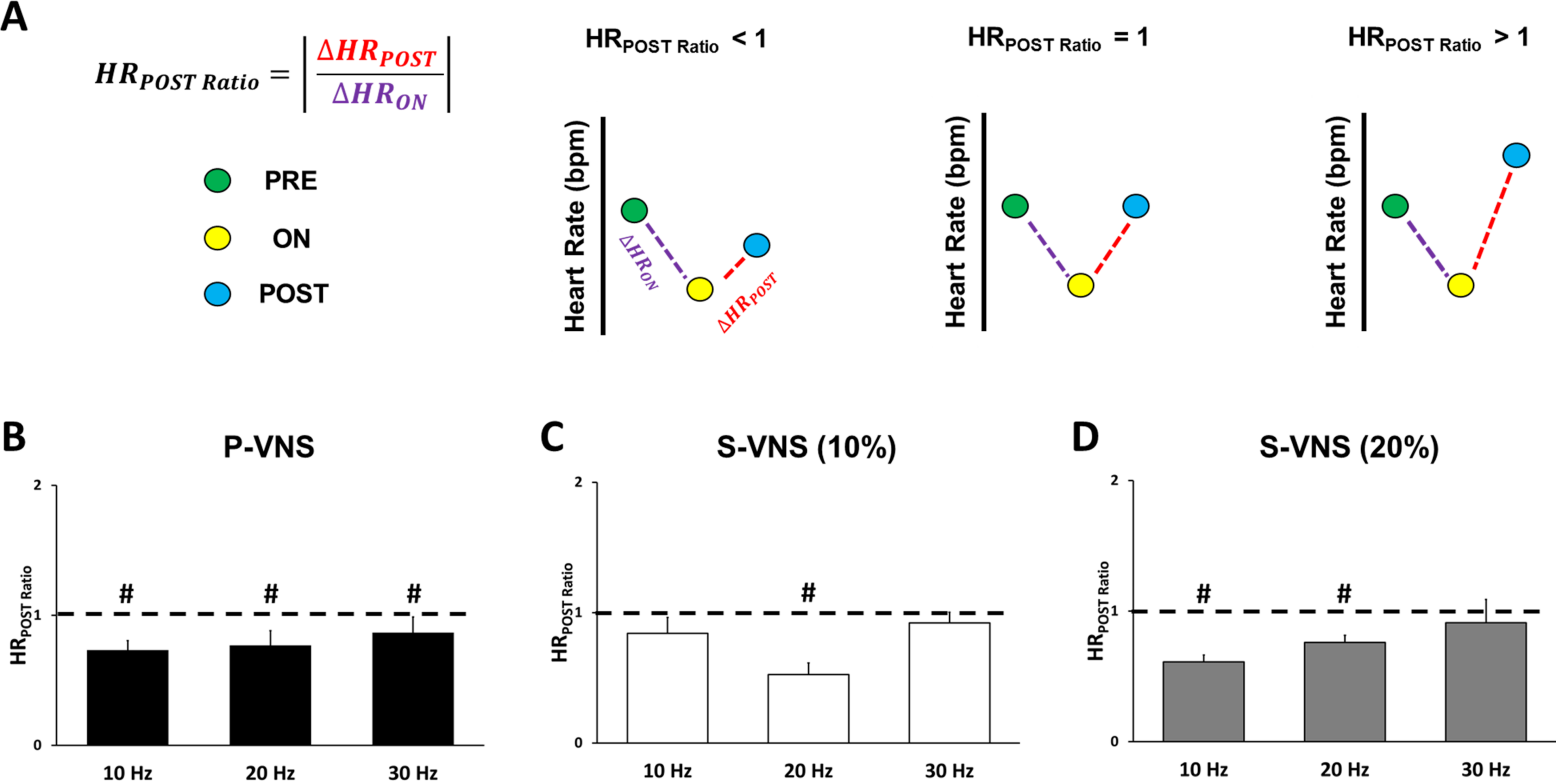
Effects of VNS FREQ on HR recovery. **(A)** Schematic representation of different scenarios of HR recovery based on the value of *HR*_*POST Ratio*_. Mean *HR*_*POST Ratio*_ values for **(B)** P-VNS, **(C)** S-VNS (10%), and **(D)** S-VNS (20%) at different FREQ. # p<0.05 compared to a mean theoretical value of 1.

To evaluate the recovery of HR to baseline values after VNS application, *HR*_*POST Ratio*_ was calculated across different FREQs. Our results from Panels B-D in Fig 6 indicate that for FREQ = 30 Hz, the HR could recover back to its **PRE** value in S-VNS protocols. However, for FREQ = 10 or 20 Hz, the *HR*_*POST Ratio*_ was less than 1, reflecting a persistent reduction in HR after the end of the VNS cycle.

## Discussion

The aim of the present study was to develop and test a new autonomic modulation stimulation protocol using stochastic stimulation (S-VNS), and compare it with traditional stimulation waveforms (P-VNS). Specifically, the evoked chronotropic response to P-VNS (delivered at a constant FREQ of 10, 20, or 30 Hz) were evaluated, and compared to S-VNS where stochastic variability was imposed in delivered FREQs with constraints for 10% or 20% around these three FREQs. The major findings of this study are as follows: (1) HR is reduced by both S-VNS and P-VNS in a FREQ-dependent manner. However, the negative chronotropic effect of S-VNS at 10 Hz and 20 Hz is significantly smaller than that of P-VNS and (2) S-VNS may acutely modulate the nonlinear relationship between short- and long-term HRV differently than P-VNS, as indicated by SD1/SD2 measurements. Overall, the results of our study suggest that acute S-VNS affects the dynamic changes in *in-vivo* rat hearts, specifically HR and HRV in a different manner, when compared to traditional P-VNS. Hence, our study further supports the notion that different modes of stimulation provides different outcomes as measured by means of change in HR. We also highly suggest further investigations into characterizing the effects and elucidating the mechanisms of S-VNS as a novel neuromodulation technique under different diseased conditions.

### The need to optimize VNS parameters

It has been well-established that an important feature of the adverse pathophysiological changes seen in HF is autonomic imbalance, characterized by sympathetic hyperactivation and parasympathetic withdrawal [29]. VNS has emerged as a promising therapy to normalize parasympathetic activation of cardiac control and restores normal levels of autonomic regulatory function. In fact, several animal studies suggest VNS exhibits both anti-arrhythmic and anti-inflammatory effects and attenuates myocardial damage and cardiac myocyte apoptosis [2,4,11]. Based on the encouraging results of the preclinical studies, several clinical VNS studies have been conducted. Both ANTHEM-HF and the subsequent extension study (ENCORE) yielded favorable and encouraging results as patients who received VNS experienced significant improvements in left ventricular (LV) structure and function, decreased HF symptom expression and decreased T-wave alternans, a measure of electrical instability [5,7]. However, other randomized clinical trials, such as the INOVATE-HF and NECTAR-HF, failed to demonstrate significant improvements in clinical outcomes owing to imperfect inclusion/exclusion criteria and sub-therapeutic VNS dosing in significant (~30–50%) of the enrolled population [8,9].

Yet despite these and other studies, there still does not exist a universally accepted published prospective stimulation paradigm, which further highlights the importance and complexity of parameter optimization. Hence, to further optimize and improve VNS efficacy, this study introduced S-VNS, a novel technique that stimulates the vagus nerve stochastically since it is clinically well-established that healthy hearts have been associated with high HRV [14].

### Effects of stochasticity on HR and HRV

Our study demonstrated a FREQ-dependent reduction in HR as a result of VNS, which was previously shown in several studies asserting that increasing the stimulation FREQ alters the rate of propagation of action potential through the vagus nerve fibers [13]. However, we also observed that the drop in HR is significantly smaller for S-VNS as compared to P-VNS, even though mean FREQ was similar for both techniques. One of the possible reasons for this is that although the same number of stimuli were applied during both stimulation techniques, stimuli during S-VNS were distributed more randomly. In P-VNS, there was a consistent number of stimuli being delivered between RR intervals, as highlighted in Fig 2.

To determine the residual functional effects occurring after cessation of VNS, we calculated *HR*_*POST Ratio*_. Our results show that the post HR of both S-VNS and P-VNS at certain FREQs did not return to baseline. This could be attributed to the fact that we applied continuous stimulation which is much longer than the typical intermittent VNS (e.g. duty cycle of 10% or 20%). Therefore, while other studies report a recovery to baseline at end of their intermittent stimulation [7,13], continuous stimulation may require more than the collected 2 minutes **POST** for the HR to return to baseline. Another possible explanation is that the cardiac effects of VNS may persist beyond the end of stimulation, and exhibit a “memory effect,” a phenomenon similar to cardiac memory. Cardiac memory is an established property of paced cardiac myocytes to reflect the influence of pacing history and adapt and respond to novel stimuli. Several experimental studies and numerical simulations have shown that memory plays a role in modulating cardiac dynamics [30,31]. Indeed, in future studies, we will examine post-VNS recovery after a longer time delay.

In this study, we performed both linear and nonlinear approaches to evaluate the effects of VNS on HRV. Poincaré plots of RR intervals is a nonlinear approach used to monitor autonomic changes by means of SD1 and SD2. Physiologically, SD1 is an index of instantaneous recording of the beat-to-beat variability and reflects primarily the parasympathetic input to the heart, while SD2 is an index of long term variability which reflects the overall variability influenced by both the parasympathetic and sympathetic contributions [25,32]. Hence, their ratio (SD1/SD2) provides a measurement between both short term and long-term variability in the RR intervals. Libbus and colleagues recently reported that patients in the ANTHEM-HF trial who received chronic, right-sided intermittent P-VNS expressed an increase in the SD1/SD2 ratio, which suggested that right-sided VNS increases the balance in favor of parasympathetic dominance over sympathetic activity [32]. While our acute P-VNS did not induce any changes in SD1/SD2 ratio in rats, our experiments demonstrated that acute continuous S-VNS significantly decreased this ratio, specifically between 10 Hz and 30 Hz. We also observed no difference in SD1 and SD2 values with increase in stimulation FREQ across **PRE**, **ON**, and **POST** for P-VNS and S-VNS (S2 Fig). This data suggests that FREQ and STOCH not only play a role in the chronotropic effects of VNS, but also in the engagement of the autonomic nervous system via relative difference in ACh release between the two VNS techniques. Nonetheless, it is worth noting that brief changes in HRV, i.e. during VNS **ON** phase, may or may not have any significant effects on clinical outcomes, and will need to be further investigated. While Poincaré maps suggest a change in autonomic activity, our ratio of SDRR to mean RR saw no significant HRV difference induced by both S-VNS and P-VNS (S1 Fig). Indeed, there are several metrics to gauge autonomic activity which include time-domain (e.g. SDNN, RMSSD,…), frequency-domain (e.g. LF, HF, LF/HF,…), and nonlinear (Poincaré, sample entropy, detrended fluctuation analysis,…) methods [33]. Although previous studies have shown a possible correlation between the different measures [33,34], an exact equivalence has not been reported. It is possible that Poincaré plots may detect abnormalities that are not as easily detectable with traditional time-domain measures, given that each parameter has different sensitivity to noise and other experimental conditions [35]. Hence, further investigations to fully uncover and understand the physiological relevance of these autonomic assessment methods are warranted in order to determine the efficacy of the different metrics in accurately quantifying autonomic activity”.

### Mechanistic insights into acute S-VNS

In the present study, we observed a smaller drop in HR and a reduction in SD1/SD2 ratio in S-VNS compared to P-VNS. From a physiological standpoint, this was a surprising finding since applying S-VNS does not necessarily correlate to stochastic release of ACh and stimulation of the muscarinic receptors of the cardiomyocytes. Rather, the efferent vagal fibers which are responsible for carrying descending information from the brain to the heart do not directly synapse with cardiomyocytes but rather with the intrinsic cardiac nervous system (ICNS), which acts as a buffer to register and send commands to the cardiomyocytes [13]. The ICNS is comprised of an intricate network of ganglia (and its neurons) that can independently act or communicate with its counterparts in the intrathoracic extracardiac, brain stem, spinal cord, and cortex to regulate intracardiac reflexes [13]. Although detailed studies will need to be performed to quantify the level of ACh release during the two stimulation protocols, we hypothesize that the observed minimal HR drop can be due to the fact that the temporal summation of ACh release is lower during S-VNS when compared to P-VNS protocol. This could be due to the fact that the instantaneous, effective FREQ of stimulation during S-VNS protocol is different (either less or more) from the corresponding FREQ of P-VNS protocol.

Phase response curves (PRC) detail how a system with its own intrinsic properties evolve with an external perturbation. The heart is an example of such dynamical systems that when given an external stimulus can cause either phase mismatch or entrainment. It has been well established that the chronotropic response to vagal stimulation is phase-dependent [36]. In fact, depending on the timing, duration, and magnitude of the vagal pulse, repetitive vagal stimulation has been shown to be able to regulate the SA cells to beat at rates that are different from its intrinsic cycle length [37]. Therefore, another possible explanation for our findings is that incorporating STOCH in our VNS may affect the level of entrainment between the vagal stimuli and the cycle length of the SA cells. Future additional studies should be conducted to understand how S-VNS may affect the intrinsic PRC.

There has been an emerging interest in using closed-loop neuro-stimulation to achieve targeted control of cardiac parameters [38]. In fact, significant developments have been made in this area based on state transition models where an algorithm optimally selects the VNS parameters to regulate instantaneous HR synchronously with cardiac cycles [39]. We hypothesize that incorporating STOCH in closed-loop VNS may potentially lead to improved therapeutic efficacy. Physiologically, the heart rhythm is not deterministic and is known to exhibit beat-to-beat variability (HRV) caused by numerous physiological factors including the influence of circadian rhythms, respiratory rhythms, temperature regulations, and others [40]. In fact, it has been associated that low HRV is a predictor for pathological cardiac conditions [16]. Hence, we hypothesize that by recording real-time changes in HRV and then integrating this “inherent stochastic feedback” into the stimulation of the VNS through STOCH may lead to a more physiological stimulation of the vagus and increase the efficacy of VNS to treat cardiac disease. This will require a fully functional, implantable, and chronic version of the S-VNS system that has real-time monitoring capabilities.

### Limitations

One of the limitations of our study is that we only investigated the differences between P-VNS and S-VNS with respect to HR and HRV, and used a healthy rat model. Furthermore, we only evaluated the acute effects. Beneficial effects have been observed when chronic P-VNS was applied in both healthy and diseased conditions [3,4,7,11]; therefore, additional chronic studies in both healthy and diseased conditions need to be performed in order to evaluate the full potential of S-VNS.

Moreover, our results suggest that S-VNS may enable some flexibility in nerve response by its ability to vary the stimulation intervals and providing a ‘pseudo-intermittent’ stimulation. Therefore, even though the same mean FREQ in both P-VNS and S-VNS were delivered, S-VNS had a smaller drop in HR. This could make S-VNS suitable for treatment of diseased conditions such as hypertension, where a controlled drop in blood pressure with a minimal change in HR are desired. This study suggests that incorporating STOCH may provide room for titration of other stimulation parameters (i.e. duty cycle and output current). In fact, in terms of treatment of brain disorders, non-periodic stimulation is currently being studied in the preclinical setting with promising results. Examples of stochastic neuromodulation techniques include but not limited to stochastic galvanic vestibular stimulation and transcranial random noise stimulation [41,42]. Therefore, there is the potential for S-VNS to induce changes in physiological parameters other than HR and HRV, making it a plausible candidate to improve current VNS treatment for epilepsy and depression, but this will require more in-depth investigation.

Furthermore, it is well accepted that the cervical vagus nerve is composed of both myelinated A and B fibers and unmyelinated C fibers [43], and our study does not address the question as to which fibers are indeed stimulated. Zagon et al have demonstrated that providing higher stimulation amplitudes, i.e. 200 µA, activates both unmyelinated and myelinated fibers in anesthetized rats [44]. Hence, this suggests that our stimulation current of 1 mA is sufficient to activate the whole thin vagal trunk of our rats. However, the discharge frequencies between afferent and efferent fibers may be different, and thus, future studies are needed to determine whether the mechanisms of both P-VNS and S-VNS occurs primarily through the afferent or efferent pathways.

Another limitation of our study is that the sequence testing was not randomized. However, we did not monotonically increase our FREQ from 10 Hz to 30 Hz. Instead the sequence order was 20 Hz, 30 Hz, and 10 Hz, but nonetheless, there may be the potential of order effects. Finally, the ECG recordings from rats were obtained with the use of general anesthesia, which is known to affect the HR of animals as compared to conscious animal ECG telemetry recordings. In fact, it has been reported that high-dose isoflurane can suppress the central cardiac parasympathetic activity in rats [45,46]. As a result, we ensured that the dosage of isoflurane administered was low (~1.5%) and remained identical across all rats throughout the duration of the study; therefore, it should not affect the interpretation of our results.

Due to the long duration of the experimental procedures, we only tested the effect of STOCH values of 10% and 20% in this present manuscript. Additional experiments will need to be performed to explore the full range of STOCH values. Furthermore, we also applied fixed values of current amplitude and pulse width. It is well accepted that VNS would elicit an immediate change in HR correlating with VNS current amplitude and pulse width [7]. Hence, while a different magnitude drop in HR value may be observed, our interpretation of the effects of incorporating STOCH should not be affected. In addition, while we only observed a difference of 2% HR drop between P-VNS and S-VNS which means extrapolating this to human may make these changes negligible, it is worth noting that the average HR of healthy rats is roughly 300–350 bpm which is about five times faster than the average resting HR of humans [46,47]. Therefore, we propose these exploratory findings to first be confirmed in larger animal studies under both control and diseased conditions before being tested in humans. Nevertheless, we plan to further evaluate the robustness and efficacy of our proposed S-VNS in chronic future studies and on non-anesthetized murine models.

## Conclusions

S-VNS induces dynamical changes in *in-vivo* rat hearts and produces minimal changes in HR compared to P-VNS. Hence, our study provides evidence that stochastic and periodic modes of stimulation induce different cardiac responses, and we suggest further detailed investigations into understanding the full therapeutic potential of S-VNS under different diseased conditions.

## Supporting information

S1 Fig Time domain HRV analysis. HRV was calculated as a ratio of the standard deviation of RR interval (SDRR) to mean RR interval (mean RR), as described previously [28]. From this approach, no significant difference was observed for all FREQs for **PRE**, **ON**, and **POST** periods for all VNS protocols.
(TIF)

S2 Fig Values of SD1 and SD2. Mean **(A)** SD1 and **(B)** SD2 values for **PRE, ON,** and **POST** across different FREQs. No significant difference in SD1 and SD2 values were observed with increase in stimulation FREQ across **PRE**, **ON**, and **POST** for P-VNS and S-VNS.
(TIF)

S3 Fig Quantitative analysis of 1-minute stabilization interval. In regards to whether 1-minute stabilization intervals were sufficient to avoid carryover effects on **PRE,** we performed the following quantitative analysis. The timeline of HR change during entire duration of an experiment is shown in Panel A for one rat. Vertical dashed lines represent the 1-minute stabilization intervals. The red squares indicate the HR before (yellow circle) and after (blue circle) 1-minute stabilization interval. As can be seen from Panel A, there are no carryover effects. To quantitatively show this, we have calculated the difference between these two HR values (ΔHR = HRyellow—HRblue). Panel B represents ΔHR for the duration of our experiments, indicating very stable preparation with negligible ΔHR < 7 bpm with respect to HR ranged from 300–450 bpm. We performed similar analysis for all our rats’ data (n = 8).
(TIF)

S4 Fig Quantitative analysis of steady state of the ‘last’ 120 seconds of recording data. In regards to whether the last 100 seconds of the 120 seconds were in steady state, we performed the following quantitative analysis: we calculated the difference between mean HR during the first and last 20 seconds (see green boxes in Panel B) of each rat’s run in our study for all **PRE**, **ON**, and **POST** datasets for all S-VNS (STOCH = 10% and 20%) and P-VNS protocols. From this quantitative analysis we observed that there was minimal difference (<12 bpm) between the first and last 20 seconds. Mean difference between the mean HR during the first and last 20 seconds for PRE, ON, and POST are shown for C) 10 Hz, D) 20 Hz, and E) 30 Hz.
(TIF)

S5 Fig Histogram distributions of P-VNS and S-VNS protocols. Representative histogram distributions of one representative rat for P-VNS and S-VNS protocols for **PRE**, **ON**, and **POST**, delivered at 20 Hz.
(TIF)

## References

[1] O’Reardon JP, Cristancho P, Peshek AD. Vagus Nerve Stimulation (VNS) and Treatment of Depression: To the Brainstem and Beyond. Psychiatry (Edgmont). Matrix Medical Communications; 2006;3: 54–63. Available: http://www.ncbi.nlm.nih.gov/pubmed/21103178 PMC2990624 21103178

[2] Xie X, Lee SW, Johnson C, Ippolito J, KenKnight BH, Tolkacheva EG. Intermittent vagal nerve stimulation alters the electrophysiological properties of atrium in the myocardial infarction rat model. 2014 36th Annual International Conference of the IEEE Engineering in Medicine and Biology Society. IEEE; 2014 pp. 1575–1578. doi: 10.1109/EMBC.2014.6943904 25570272 10.1109/EMBC.2014.6943904

[3] Lee SW, Li Q, Libbus I, Xie X, KenKnight BH, Garry MG, et al Chronic cyclic vagus nerve stimulation has beneficial electrophysiological effects on healthy hearts in the absence of autonomic imbalance. Physiol Rep. 2016;4: e12786 doi: 10.14814/phy2.12786 27173672 10.14814/phy2.12786 PMC4873636

[4] Annoni EM, Xie X, Lee SW, Libbus I, KenKnight BH, Osborn JW, et al Intermittent electrical stimulation of the right cervical vagus nerve in salt-sensitive hypertensive rats: effects on blood pressure, arrhythmias, and ventricular electrophysiology. Physiol Rep. 2015;3 doi: 10.14814/phy2.12476 26265746 10.14814/phy2.12476 PMC4562562

[5] Premchand RK, Sharma K, Mittal S, Monteiro R, Dixit S, Libbus I, et al Extended Follow-Up of Patients with Heart Failure Receiving Autonomic Regulation Therapy in the ANTHEM-HF Study. J Card Fail. 2015; doi: 10.1016/j.cardfail.2015.11.002 26576716 10.1016/j.cardfail.2015.11.002

[6] Olshansky B. Electrical Stimulation of the Vagus Nerve for Chronic Heart Failure: Is It Time to Pull the Plug? J Card Fail. 2016;22: 643–645. doi: 10.1016/j.cardfail.2016.05.005 27233952 10.1016/j.cardfail.2016.05.005

[7] Nearing BD, Libbus I, Amurthur B, KenKnight BH, Verrier RL. Autonomic regulation therapy suppresses quantitative T-wave alternans and improves baroreflex sensitivity in patients with heart failure enrolled in the ANTHEM-HF study. Hear Rhythm. 2016;13: 721–728. doi: 10.1016/j.hrthm.2015.11.030 26601770 10.1016/j.hrthm.2015.11.030

[8] Zannad F, De Ferrari GM, Tuinenburg AE, Wright D, Brugada J, Butter C, et al Chronic vagal stimulation for the treatment of low ejection fraction heart failure: results of the NEural Cardiac TherApy foR Heart Failure (NECTAR-HF) randomized controlled trial. Eur Heart J. 2015;36: 425–433. doi: 10.1093/eurheartj/ehu345 25176942 10.1093/eurheartj/ehu345 PMC4328197

[9] Gold MR, Van Veldhuisen DJ, Hauptman PJ, Borggrefe M, Kubo SH, Lieberman RA, et al Vagus Nerve Stimulation for the Treatment of Heart Failure. J Am Coll Cardiol. 2016;68: 149–158. doi: 10.1016/j.jacc.2016.03.525 27058909 10.1016/j.jacc.2016.03.525

[10] Yoo PB, Liu H, Hincapie JG, Ruble SB, Hamann JJ, Grill WM. Modulation of heart rate by temporally patterned vagus nerve stimulation in the anesthetized dog. Physiol Rep. 2016;4: e12689 doi: 10.14814/phy2.12689 26811057 10.14814/phy2.12689 PMC4760392

[11] Li M, Zheng C, Sato T, Kawada T, Sugimachi M, Sunagawa K. Vagal nerve stimulation markedly improves long-term survival after chronic heart failure in rats. Circulation. 2004;109: 120–4. doi: 10.1161/01.CIR.0000105721.71640.DA 14662714 10.1161/01.CIR.0000105721.71640.DA

[12] Kong S-S, Liu J-J, Hwang T-C, Yu X-J, Zhao M, Zhao M, et al Optimizing the Parameters of Vagus Nerve Stimulation by Uniform Design in Rats with Acute Myocardial Infarction. Tache Y, editor. PLoS One. Public Library of Science; 2012;7: e42799 doi: 10.1371/journal.pone.0042799 23189120 10.1371/journal.pone.0042799 PMC3506552

[13] Ardell JL, Rajendran PS, Nier HA, KenKnight BH, Armour JA. Central-peripheral neural network interactions evoked by vagus nerve stimulation: functional consequences on control of cardiac function. Am J Physiol Heart Circ Physiol. 2015;309: H1740–52. doi: 10.1152/ajpheart.00557.2015 26371171 10.1152/ajpheart.00557.2015 PMC4666982

[14] Malik M. Heart Rate Variability. Ann Noninvasive Electrocardiol. Blackwell Publishing Ltd; 1996;1: 151–181. doi: 10.1111/j.1542-474X.1996.tb00275.x

[15] Hori M, Okamoto H. Heart rate as a target of treatment of chronic heart failure. J Cardiol. 2012;60: 86–90. doi: 10.1016/j.jjcc.2012.06.013 22920717 10.1016/j.jjcc.2012.06.013

[16] Balasubramanian K, Harikumar K, Nagaraj N, Pati S. Vagus Nerve Stimulation Modulates Complexity of Heart Rate Variability Differently during Sleep and Wakefulness. Ann Indian Acad Neurol. Wolters Kluwer—Medknow Publications; 2017;20: 403–407. doi: 10.4103/aian.AIAN_148_17 29184345 10.4103/aian.AIAN_148_17 PMC5682746

[17] Shen MJ, Zipes DP. Role of the autonomic nervous system in modulating cardiac arrhythmias. Circ Res. 2014;114: 1004–21. doi: 10.1161/CIRCRESAHA.113.302549 24625726 10.1161/CIRCRESAHA.113.302549

[18] Kapa S, DeSimone C V, Asirvatham SJ. Innervation of the heart: An invisible grid within a black box. Trends Cardiovasc Med. NIH Public Access; 2016;26: 245–57. doi: 10.1016/j.tcm.2015.07.001 26254961 10.1016/j.tcm.2015.07.001 PMC4706824

[19] Harvey RD, Belevych AE. Muscarinic regulation of cardiac ion channels. Br J Pharmacol. 2003;139: 1074–1084. doi: 10.1038/sj.bjp.0705338 12871825 10.1038/sj.bjp.0705338 PMC1573933

[20] Lund C, Kostov H, Blomskjøld B, Nakken KO. Efficacy and tolerability of long-term treatment with vagus nerve stimulation in adolescents and adults with refractory epilepsy and learning disabilities. Seizure. 2011;20: 34–37. doi: 10.1016/j.seizure.2010.10.002 21035358 10.1016/j.seizure.2010.10.002

[21] Samniang B, Shinlapawittayatorn K, Chunchai T, Pongkan W, Kumfu S, Chattipakorn SC, et al Vagus Nerve Stimulation Improves Cardiac Function by Preventing Mitochondrial Dysfunction in Obese-Insulin Resistant Rats. Sci Rep. Nature Publishing Group; 2016;6: 19749 doi: 10.1038/srep19749 26830020 10.1038/srep19749 PMC4735283

[22] Buschman HP, Storm CJ, Duncker DJ, Verdouw PD, van der Aa HE, van der Kemp P. Heart Rate Control Via Vagus Nerve Stimulation. Neuromodulation Technol Neural Interface. 2006;9: 214–220. doi: 10.1111/j.1525-1403.2006.00062.x 22151709 10.1111/j.1525-1403.2006.00062.x

[23] Muppidi S, Gupta PK, Vernino S. Reversible right vagal neuropathy. Neurology. American Academy of Neurology; 2011;77: 1577–9. doi: 10.1212/WNL.0b013e318233b3a2 21975204 10.1212/WNL.0b013e318233b3a2 PMC3198976

[24] Tarvainen MP, Niskanen J-P, Lipponen JA, Ranta-aho PO, Karjalainen PA. Kubios HRV–Heart rate variability analysis software. Comput Methods Programs Biomed. 2014;113: 210–220. doi: 10.1016/j.cmpb.2013.07.024 24054542 10.1016/j.cmpb.2013.07.024

[25] Brennan M, Palaniswami M, Kamen P. Do existing measures of Poincare plot geometry reflect nonlinear features of heart rate variability? IEEE Trans Biomed Eng. 2001;48: 1342–1347. doi: 10.1109/10.959330 11686633 10.1109/10.959330

[26] Parker P, Celler BG, Potter EK, McCloskey DI. Vagal stimulation and cardiac slowing. J Auton Nerv Syst. 1984;11: 226–31. Available: http://www.ncbi.nlm.nih.gov/pubmed/6491162 6491162 10.1016/0165-1838(84)90080-8

[27] Berntson GG, Quigley KS, Fabro VJ, Cacioppo JT. Vagal stimulation and cardiac chronotropy in rats. J Auton Nerv Syst. 1992;41: 221–6. Available: http://www.ncbi.nlm.nih.gov/pubmed/1289386 1289386 10.1016/0165-1838(92)90062-l

[28] McIntyre SD, Kakade V, Mori Y, Tolkacheva EG. Heart rate variability and alternans formation in the heart: The role of feedback in cardiac dynamics. J Theor Biol. 2014;350: 90–7. doi: 10.1016/j.jtbi.2014.02.015 24576615 10.1016/j.jtbi.2014.02.015

[29] De Ferrari GM, Crijns HJGM, Borggrefe M, Milasinovic G, Smid J, Zabel M, et al Chronic vagus nerve stimulation: a new and promising therapeutic approach for chronic heart failure. Eur Heart J. 2011;32: 847–55. doi: 10.1093/eurheartj/ehq391 21030409 10.1093/eurheartj/ehq391

[30] Tolkacheva EG. The rate- and species-dependence of short-term memory in cardiac myocytes. J Biol Phys. Springer; 2007;33: 35–47. doi: 10.1007/s10867-007-9040-5 19669551 10.1007/s10867-007-9040-5 PMC2646391

[31] Libbus I, Rosenbaum DS. Remodeling of cardiac repolarization: mechanisms and implications of memory. Card Electrophysiol Rev. 2002;6: 302–10. Available: http://www.ncbi.nlm.nih.gov/pubmed/12114856 12114856 10.1023/a:1016349613464

[32] Libbus I, Nearing BD, Amurthur B, KenKnight BH, Verrier RL. Quantitative evaluation of heartbeat interval time series using Poincaré analysis reveals distinct patterns of heart rate dynamics during cycles of vagus nerve stimulation in patients with heart failure. J Electrocardiol. 2017; doi: 10.1016/j.jelectrocard.2017.06.007 28625397 10.1016/j.jelectrocard.2017.06.007

[33] Shaffer F, Ginsberg JP. An Overview of Heart Rate Variability Metrics and Norms. Front Public Heal. Frontiers; 2017;5: 258 doi: 10.3389/fpubh.2017.00258 29034226 10.3389/fpubh.2017.00258 PMC5624990

[34] Guzik P, Piskorski J, Krauze T, Schneider R, Wesseling KH, Wykretowicz A, et al Correlations between the Poincaré Plot and Conventional Heart Rate Variability Parameters Assessed during Paced Breathing. J Physiol Sci. PHYSIOLOGICAL SOCIETY OF JAPAN; 2007;57: 63–71. doi: 10.2170/physiolsci.RP005506 17266795 10.2170/physiolsci.RP005506

[35] Stapelberg NJC, Neumann DL, Shum DHK, McConnell H, Hamilton-Craig I. The sensitivity of 38 heart rate variability measures to the addition of artifact in human and artificial 24-hr cardiac recordings. Ann Noninvasive Electrocardiol. 2018;23: e12483 doi: 10.1111/anec.12483 28670841 10.1111/anec.12483 PMC7313264

[36] Abramovich-Sivan S, Akselrod S. A Phase Response Curve Based Model: Effect of Vagal and Sympathetic Stimulation and Interaction on a Pacemaker Cell. J Theor Biol. 1998;192: 567–579. doi: 10.1006/jtbi.1998.0684 9680725 10.1006/jtbi.1998.0684

[37] Yang T, Jacobstein MD, Levy MN. Synchronization of automatic cells in S-A node during vagal stimulation in dogs. Am J Physiol. 1984;246: H585–91. Available: http://www.ncbi.nlm.nih.gov/pubmed/6720914 doi: 10.1152/ajpheart.1984.246.4.H585 6720914 10.1152/ajpheart.1984.246.4.H585

[38] Ugalde HR, Le Rolle V, Bel A, Bonnet J-L, Andreu D, Mabo P, et al On-off closed-loop control of vagus nerve stimulation for the adaptation of heart rate. 2014 36th Annual International Conference of the IEEE Engineering in Medicine and Biology Society. IEEE; 2014 pp. 6262–6265. doi: 10.1109/EMBC.2014.6945060 25571428 10.1109/EMBC.2014.6945060

[39] Romero Ugalde HM, Le Rolle V, Bonnet J-L, Henry C, Mabo P, Carrault G, et al Closed-loop vagus nerve stimulation based on state transition models. IEEE Trans Biomed Eng. 2017; 1–1. doi: 10.1109/TBME.2017.2759667 28991730 10.1109/TBME.2017.2759667

[40] Dvir H, Zlochiver S. Stochastic cardiac pacing increases ventricular electrical stability—a computational study. Biophys J. The Biophysical Society; 2013;105: 533–42. doi: 10.1016/j.bpj.2013.06.012 23870274 10.1016/j.bpj.2013.06.012 PMC3714878

[41] van der Groen O, Wenderoth N. Random Noise Stimulation of the Cortex: Stochastic Resonance Enhances Central Mechanisms of Perception. Brain Stimul. 2017;10: e4 doi: 10.1016/j.brs.2016.11.030 10.1523/JNEUROSCI.4519-15.2016 PMC6601807 27170126

[42] Goel R, Kofman I, Jeevarajan J, De Dios Y, Cohen HS, Bloomberg JJ, et al Using Low Levels of Stochastic Vestibular Stimulation to Improve Balance Function. Chacron MJ, editor. PLoS One. Public Library of Science; 2015;10: e0136335 doi: 10.1371/journal.pone.0136335 26295807 10.1371/journal.pone.0136335 PMC4546608

[43] Yuan H, Silberstein SD. Vagus Nerve and Vagus Nerve Stimulation, a Comprehensive Review: Part I. Headache J Head Face Pain. 2016;56: 71–78. doi: 10.1111/head.12647 26364692 10.1111/head.12647

[44] Zagon A, Kemeny AA. Slow hyperpolarization in cortical neurons: a possible mechanism behind vagus nerve simulation therapy for refractory epilepsy? Epilepsia. 2000;41: 1382–9. Available: http://www.ncbi.nlm.nih.gov/pubmed/11077451 11077451 10.1111/j.1528-1157.2000.tb00113.x

[45] Toader E, Cividjian A, Quintin L. Isoflurane suppresses central cardiac parasympathetic activity in rats: a pilot study. Minerva Anestesiol. 2011;77: 142–6. Available: http://www.ncbi.nlm.nih.gov/pubmed/21150849 21150849

[46] Yang C-F, Yu-Chih Chen M, Chen T-I, Cheng C-F. Dose-dependent effects of isoflurane on cardiovascular function in rats. Tzu Chi Med J. No longer published by Elsevier; 2014;26: 119–122. doi: 10.1016/J.TCMJ.2014.07.005

[47] Azar T, Sharp J, Lawson D. Heart rates of male and female Sprague-Dawley and spontaneously hypertensive rats housed singly or in groups. J Am Assoc Lab Anim Sci. American Association for Laboratory Animal Science; 2011;50: 175–84. Available: http://www.ncbi.nlm.nih.gov/pubmed/21439210 21439210 PMC3061417

