# Supplementary figures and images for: Stochastic vagus nerve stimulation affects acute heart rate dynamics in rats
Source: PLoS One. 2018 Mar 28;13(3):e0194910. doi: 10.1371/journal.pone.0194910 (PMC5874066; doi:10.1371/journal.pone.0194910)

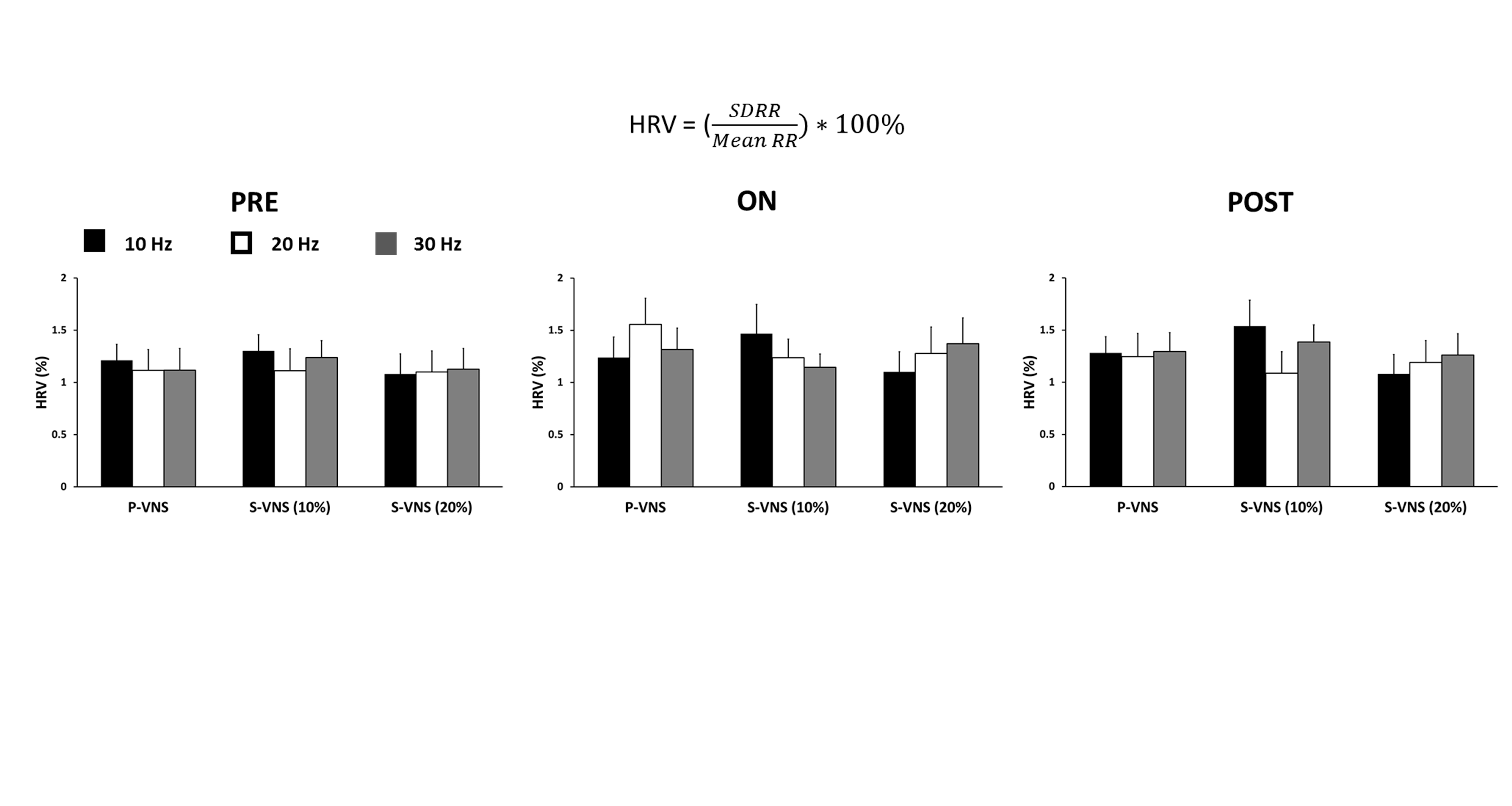

Supplement: S1 Fig — HRV was calculated as a ratio of the standard deviation of RR interval (SDRR) to mean RR interval (mean RR), as described previously [28]. From this approach, no significant difference was observed for all FREQs for PRE, ON, and POST periods for all VNS protocols. (TIF) [file pone.0194910.s001.tif]

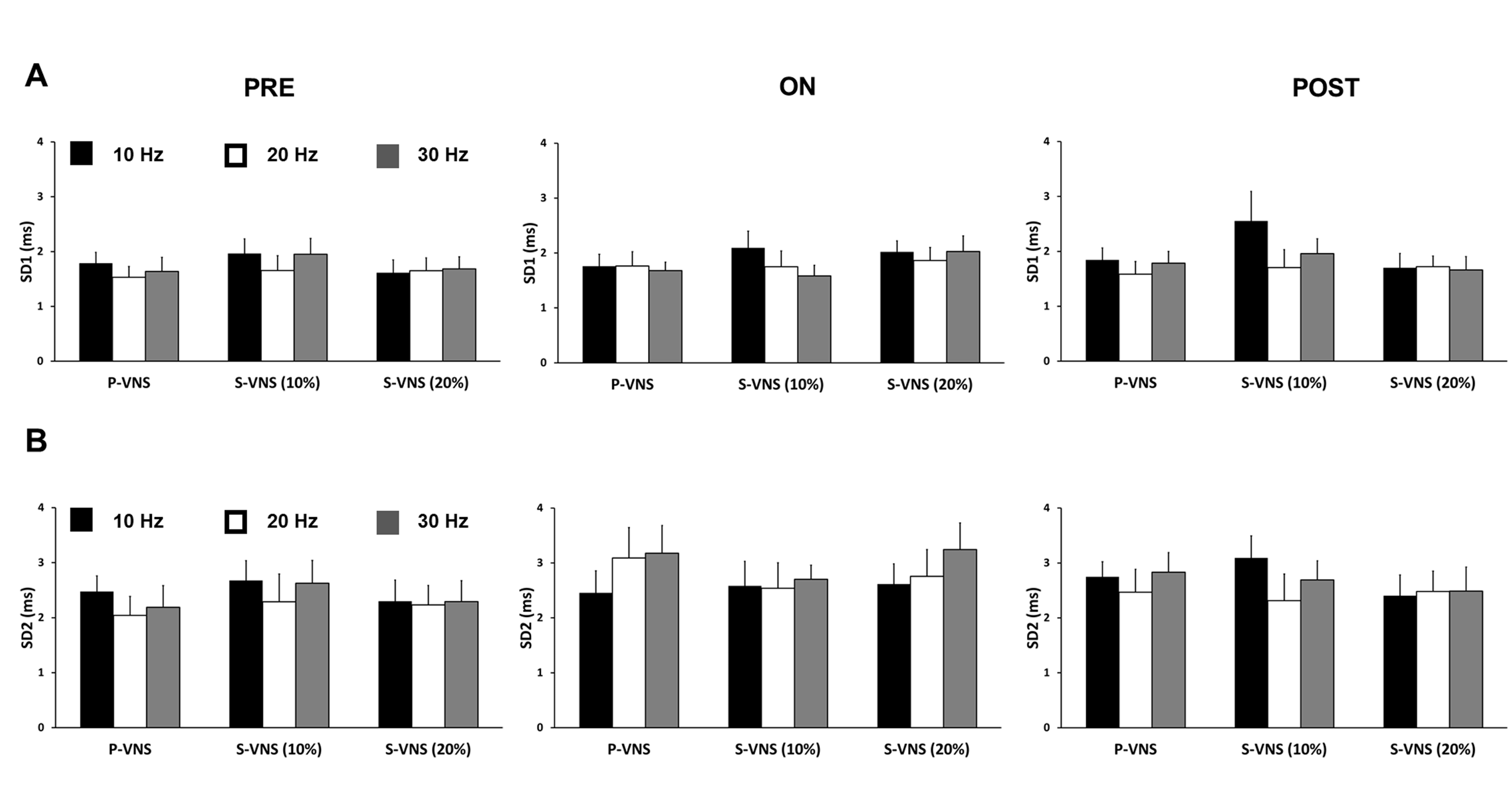

Supplement: S2 Fig — Mean (A) SD1 and (B) SD2 values for PRE, ON, and POST across different FREQs. No significant difference in SD1 and SD2 values were observed with increase in stimulation FREQ across PRE, ON, and POST for P-VNS and S-VNS. (TIF) [file pone.0194910.s002.tif]

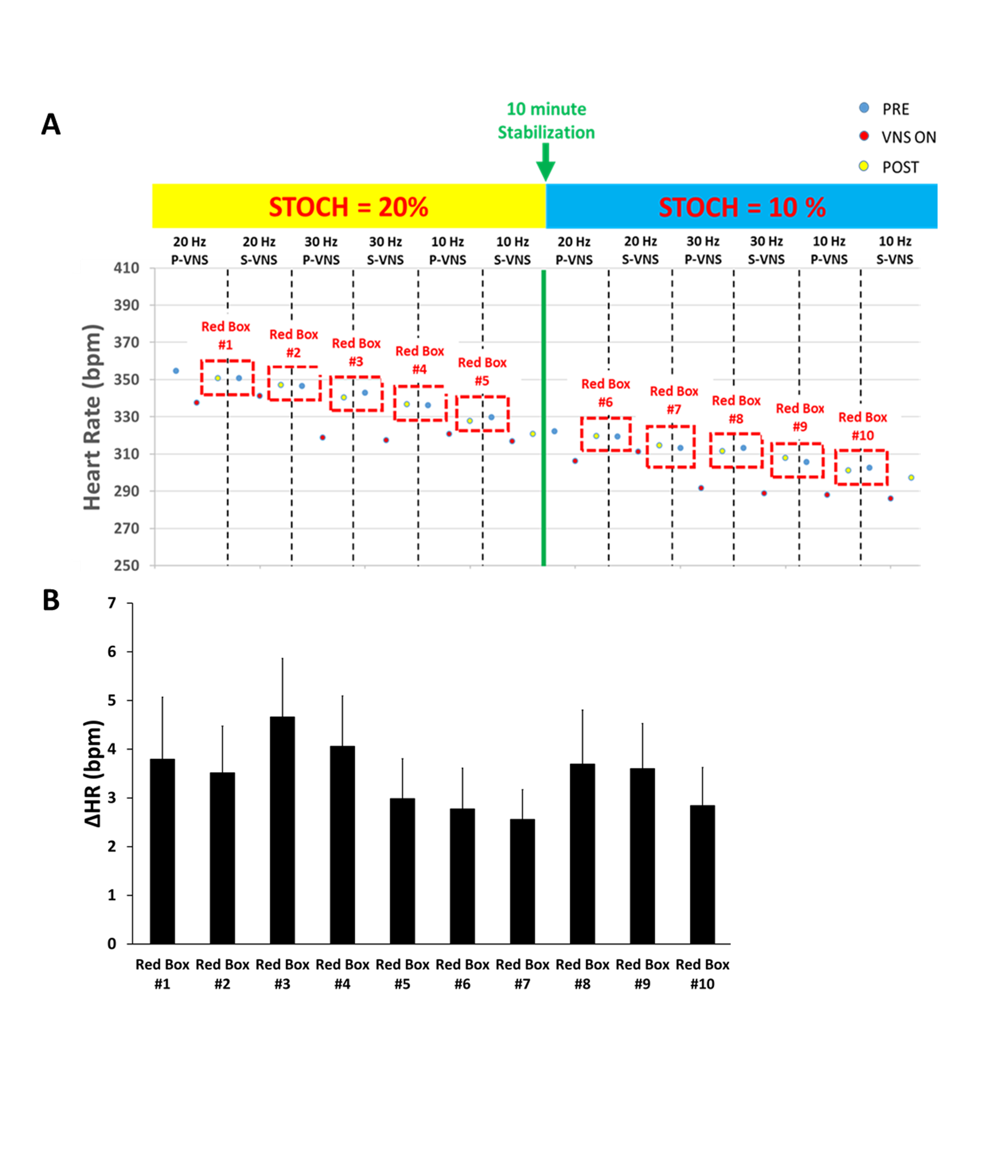

Supplement: S3 Fig — In regards to whether 1-minute stabilization intervals were sufficient to avoid carryover effects on PRE, we performed the following quantitative analysis. The timeline of HR change during entire duration of an experiment is shown in Panel A for one rat. Vertical dashed lines represent the 1-minute stabilization intervals. The red squares indicate the HR before (yellow circle) and after (blue circle) 1-minute stabilization interval. As can be seen from Panel A, there are no carryover effects. To quantitatively show this, we have calculated the difference between these two HR values (ΔHR = HRyellow—HRblue). Panel B represents ΔHR for the duration of our experiments, indicating very stable preparation with negligible ΔHR < 7 bpm with respect to HR ranged from 300–450 bpm. We performed similar analysis for all our rats’ data (n = 8). (TIF) [file pone.0194910.s003.tif]

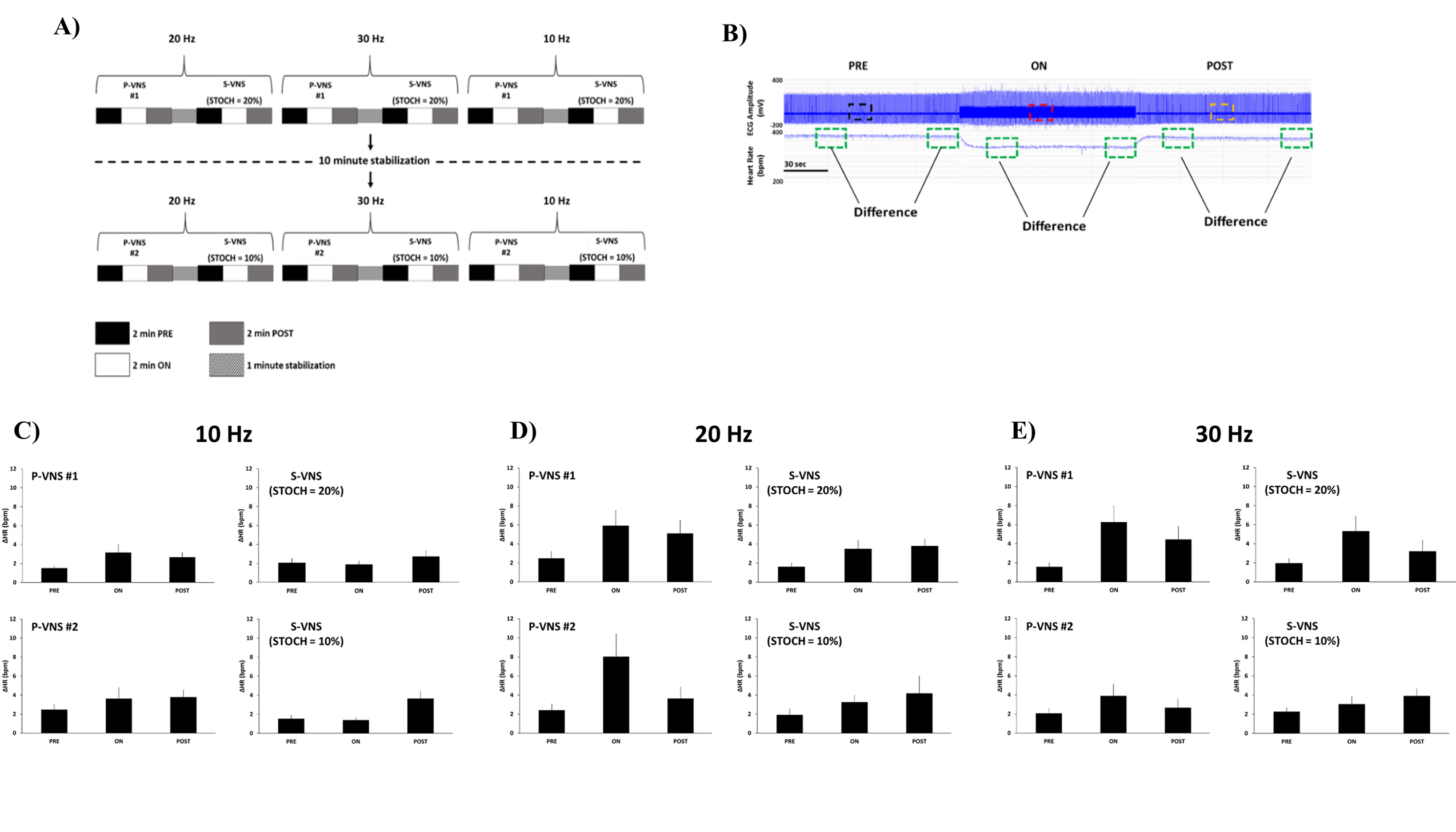

Supplement: S4 Fig — In regards to whether the last 100 seconds of the 120 seconds were in steady state, we performed the following quantitative analysis: we calculated the difference between mean HR during the first and last 20 seconds (see green boxes in Panel B) of each rat’s run in our study for all PRE, ON, and POST datasets for all S-VNS (STOCH = 10% and 20%) and P-VNS protocols. From this quantitative analysis we observed that there was minimal difference (<12 bpm) between the first and last 20 seconds. Mean difference between the mean HR during the first and last 20 seconds for PRE, ON, and POST are shown for C) 10 Hz, D) 20 Hz, and E) 30 Hz. (TIF) [file pone.0194910.s004.tif]

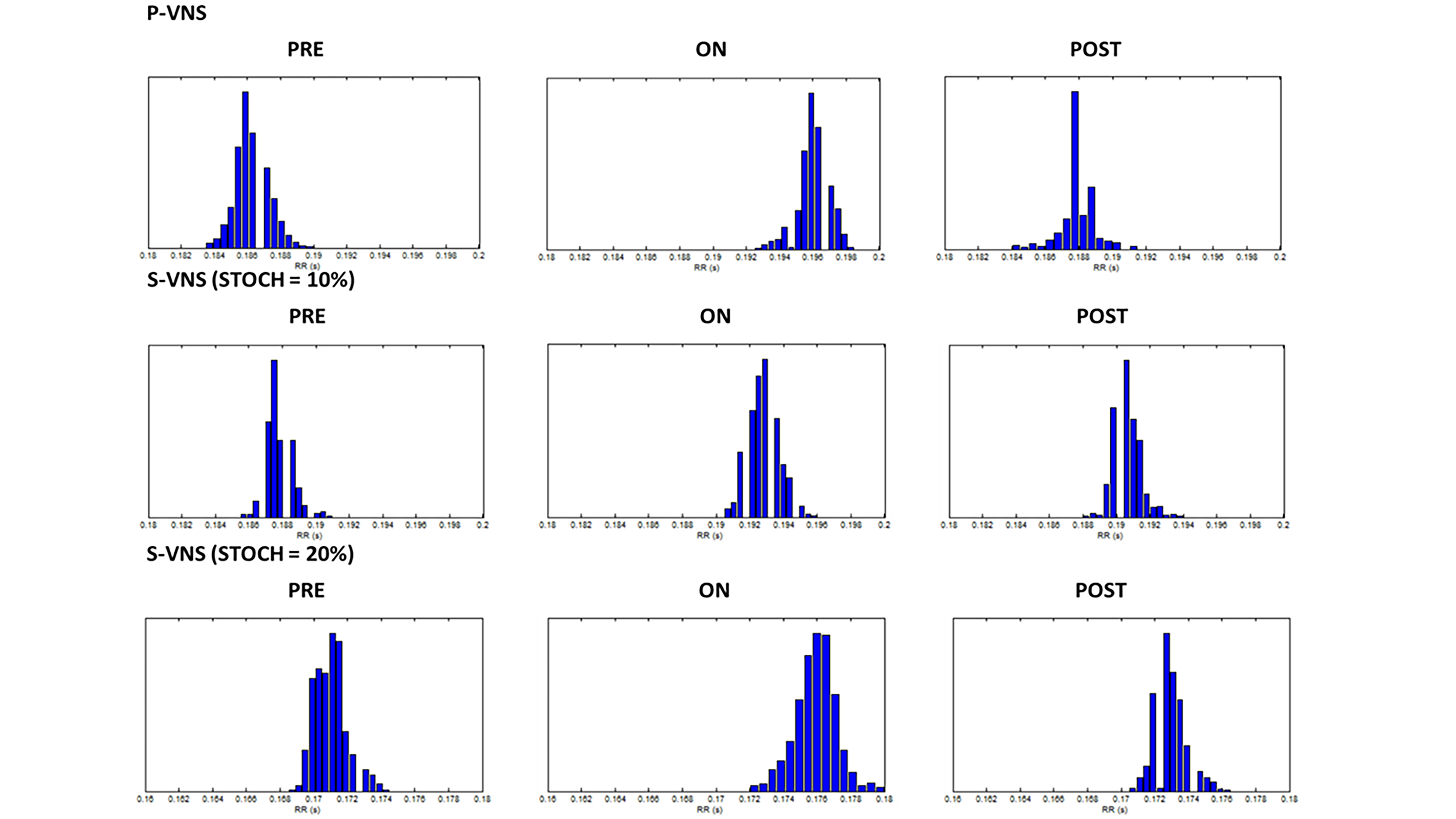

Supplement: S5 Fig — Representative histogram distributions of one representative rat for P-VNS and S-VNS protocols for PRE, ON, and POST, delivered at 20 Hz. (TIF) [file pone.0194910.s005.tif]
